# Nursing students’ experience with clinical placement in nursing homes: a focus group study

**DOI:** 10.1186/s12912-021-00690-4

**Published:** 2021-09-06

**Authors:** Kristin Laugaland, Kari Kaldestad, Elin Espeland, Brendan McCormack, Kristin Akerjordet, Ingunn Aase

**Affiliations:** 1 SHARE – Centre for Resilience in Healthcare, Faculty of Health Sciences, University of Stavanger, Kjell Arholms Gate 41, 4036 Stavanger, Norway; 2 Faculty of Health Sciences, University of Stavanger, Stavanger, Norway; 3 School of Health Sciences, Queen Margaret University, Edinburgh, UK

**Keywords:** Nursing home, Clinical placement, First-year nursing students, Placement experiences

## Abstract

**Background:**

A renewed interest in nursing homes as clinical placement settings for nursing students has been prompted by the growing healthcare needs of an ageing population. However, if future nurses are to be enthusiastic about working in this healthcare context, it is essential that higher education institutions that educate nurses and nursing homes that provide placement experiences to students do so with a supportive, positive, and enriched approach.

**Methods:**

To explore first-year nursing students’ placement experience in nursing homes, we conducted an exploratory qualitative study in three city-based nursing homes in western Norway. Thirteen first-year nursing students participated in the study. Three focus group interviews were conducted to explore the students’ placement experiences. Data were analysed using thematic analysis. The findings were reported using the Standards for Reporting Qualitative Research (SRQR).

**Results:**

The analysis describes five themes relating to first-year students’ placement experience in nursing homes; (1) variations in utility of pre-placement orientation and welcome at placement site; (2) a challenging learning environment; (3) spending considerable placement time with non-registered nurses; (4) considerable variability in supervision practices; and (5) a vulnerable and demanding student role.

**Conclusions:**

The research provides insight into the contextual characteristics encountered by first-year students that influence the quality of their placement experiences. Consequently, these characteristics impede access to important role models who lend support to a student’s growth and professional development, preventing full utilisation of the learning potential offered in nursing homes. Hence, we propose that targeted efforts are warranted to foster positive placement experiences and enhance students’ clinical education in nursing homes.

**Supplementary Information:**

The online version contains supplementary material available at 10.1186/s12912-021-00690-4.

## Background

There is a growing recognition that nursing students need more exposure to clinical practice education and learning experiences in primary healthcare settings, such as nursing homes [1]. The renewed interest in nursing homes as clinical placement settings has been prompted by the growing population of older people who live with increasingly complex long-term conditions and palliative care needs [1]. These changes in the patterns of healthcare need intensify the requirement for highly qualified nursing staff to work in nursing homes with older people. Moreover, although the need for nursing competence is increasing, an anticipated shortage of nurses with an advanced degree as specialists in the care of older people or gerontology is imminent [2]. Hence, university nursing programmes play a key role in preparing a workforce to meet future healthcare needs, especially those associated with an ageing population [3].

Traditionally, nursing homes represent the nursing students’ first placement in nurse education programmes [4]. Nursing home placements are often provided to foster learning about the fundamentals of nursing care and instil positive attitudes about older people [5, 6]. However, findings from previous studies report that nursing students view aged care as a negative and unattractive career choice in a nursing home context considered boring, depressing and of low status [1]. Providing the first-year student with a supportive and enriched placement experience is considered important in encouraging new graduate nurses to view aged care in nursing homes as a viable career option, aiding recruitment, and retention to this sector of healthcare [7, 8].

Nursing homes are often associated with and described as marginal learning environments because of the high number of residents with complex and comprehensive care needs, low staffing, and a less qualified workforce [8]. Hence, lack of resources and capacity to mentor and support students’ learning in nursing homes is the most frequently mentioned barrier in developing and utilising clinical placements in these settings [9, 10]. At the same time nursing homes provide ideal conditions for student learning, offering learning experiences of caring for a frail older population. Residents in nursing homes are more medically stable and can tolerate the extra time required by students to complete assessments, perform interventions, and provide person-centred care [3]. Hence, nursing homes can serve as rich educational sites that provide the students with positive and enriched learning experiences.

Various models of clinical education exist internationally with some variation across countries [11]. The predominant clinical supervision model for nursing education in Norway and elsewhere in Europe is the preceptorship model [12] in which students work with clinical staff but are mentored by a registered nurse and supported by a university-based nurse educator. Within this tripartite relationship the nurse educator is responsible for coordinating the students’ learning, supporting the integration of theoretical and practical learning, and engaging in the continuous assessment of the students’ progression. However, even within such a coordinated tripartite clinical placement model some key elements have been identified for successful student practice learning experiences [11], including, comprehensive orientation, committed academic/service partnership, effective supervision, supportive staff, nurse educators who are knowledgeable and enthusiastic about aged care, the use of creative and innovative clinical teaching strategies, and students experiencing high-quality nursing care [4, 13, 14].

Student learning in nursing homes is influenced by the interaction of a variety of individual factors (e.g. students’ characteristics, nurse educator and mentor variables), the nursing home environment (e.g. pedagogical atmosphere), and relational aspects (e.g. supportive relationships) [15]. Among students’ characteristics, age when entering nursing studies, ethnicity, and earlier work experience in healthcare services are found to affect students’ experience of learning during clinical placements in nursing homes. However, above all the supervisory relationship between the student and the registered nurse (RN) mentor is found to be of upmost importance in influencing a student’s placement and learning experience [16, 17].

A review of nursing students’ experiences with nursing home placements found that students feel unprepared for the realities of the nursing home environment and that the practice sites were unprepared for them. This was found to be the case particularly for first-year students, who often experienced being overwhelmed in nursing home placement settings [3]. Moreover, when assigned to nursing homes for clinical placement, nursing students report spending considerable time with unlicensed careers, leading to a lack of role models, lack of feedback from RNs, and missed learning opportunities [3]. Consequently, nursing students’ lack of insight into the RN role alongside their recognition of the RN’s managerial role of working in nursing homes (e.g. the systematic shifts in the RN’s role away from hands-on nursing to a more administrative focus) restricts their learning with regard to gerontological nursing [17] and contributes to reluctance in envisioning a nursing career in a nursing home setting [18]. Indeed, a survey of Norwegian nursing students on placement in nursing homes assessed the clinical learning environment more negatively than hospital placements in nearly all dimensions [19].

In general, students have described nursing home placements as emotionally demanding while feeling insecure, scared, nervous, and intimidated when working with older people [20]. Additionally, many students enter nursing home placements with a preconceived idea that learning opportunities are suboptimal in this setting compared with hospital settings. Likewise, students’ expectations of the ideals of person-centred care do not always meet the realities encountered [3].

Although the nursing home sector is forecasted to grow in importance as a site for undergraduate students, relatively little research has been undertaken to qualitatively explore students’ placement experiences in this clinical setting [3]. To address this knowledge gap, this study aimed to explore overall placement experiences of first-year nursing students in nursing homes during clinical practice education.

## Methods

### Design and setting

This study adopted an exploratory qualitative design [21]. Focus group interviews were used to explore the students’ placement experiences. The focus group method emphasises group interaction and discussions which, it is argued, yield rich and informative data [22]. Eligible participants were enrolled in a 3-year bachelor’s programme in nursing covering 180 European Credit Transfer System points. The study was conducted in three publicly funded nursing homes situated within a city-based municipality in western Norway. Standards for Reporting Qualitative Research guidelines [23] were used for this study.

### Participants

Recruitment was based on a purposive sampling strategy to yield insights and in-depth understanding from the target group [24]. First-year students on placement in the enrolled study sites (nursing homes) were invited to participate. The nursing home placements involved 8 weeks of obligatory placement for the students enrolled in the study. Invitations to participate were e-mailed to eligible participants during the student’s placement period with information about the study. Of the 45 students invited, 17 consented to participate. Four participants dropped out of the study prior to the focus group interviews owing to sickness or because they were absent on the day of the scheduled interview. Three focus group interviews (i.e. groups A, B and C) were held, one in each nursing home, with a total of 13 first-year nursing students. Focus groups A and B comprised four participants and focus group C comprised five participants. All participants were female students aged 20–23 years. Three of the participants had earlier work experience in healthcare services prior to placement. All participants were supervised by an RN mentor, and some of the students were supervised in peer learning. The students were on placement in various types of nursing homes including short-term, long-term, dementia, and rehabilitation units.

### Data collection

The focus group interviews, conducted at the placement sites during the students last week of their placement, lasted an average of 90 min. The interviews were conducted by the first and last author (KL and IA), who represent experienced qualitative researchers with backgrounds in nursing and education. One researcher moderated the content of the discussions by means of a semi-structured interview guide while the other assisted, took field notes, observed the interaction within the groups, and occasionally followed up with additional questions and clarifications. The interview guide addressed themes such as pre-placement orientation, supervision, assessment, collaboration, learning environment, and overall placement experience (see Supplementary file 1). The data were collected in March 2019.

### Data analysis

All interviews were recorded using a digital recorder and were then transcribed verbatim. The analytical approach followed Braun and Clarke’s framework for thematic analyses [25], a flexible approach for analysing qualitative data that searches for themes within data. Hence, the thematic analysis was guided by the research question and followed the six phases described by Braun and Clarke [25]: (1) becoming familiar with the data, (2) generating initial codes, (3) searching for themes, (4) reviewing themes, (5) defining and naming themes, and (6) producing the report. Four of the authors (KL, KK, EE, and IA) independently read the interviews to garner a general impression and become familiar with the transcripts. Summaries of overall impressions were shared among the research team whereby different perspectives of data were explored. Transcripts were then coded manually by the first and last author (KL and IA), highlighting meaning units relevant to the research questions in order to delineate patterns. The coded data were then sorted into potential recurring themes covering students’ experiences with placement in nursing homes. Four of the co-authors (KL, KK, EE, and IA) met to discuss and reach consensus by reviewing, modifying, and making final refinements to the themes and potential sub-themes.

### Ethical considerations

All methods were carried out in accordance with relevant guidelines and regulations. The study was approved by the Norwegian Centre for Research Data (2018/61309 and 489,776) and is exempted from ethical approval from the Norwegian Regional Committees for Medical and Health Research Ethics since no health information or patient data is registered. Informed consent was obtained from all the participants involved in the study and the students were informed about the right to withdraw from the study at any time. The students were made aware that participation or non-participation would not affect other aspects of their placement period or give them any advantages or disadvantages during their education programme. To ensure confidentiality, any identifying information about participants such as names and location was removed from the interview transcripts. In addition, numbered identifiers were randomly assigned to each of the participants and their focus group designation (e.g. P1, FGA stands for participant 1, focus group A). To protect the anonymity of the participants and the educational institution, participant characteristics are not elaborated in the paper.

## Results

The analyses identified five themes relating to first-year nursing students’ overall placement experiences when assigned to nursing homes for clinical practice education: (1) variations in utility of pre-placement orientation and welcome at the placement site; (2) a challenging learning environment; (3) spending considerable placement time with non-registered nurses; (4) considerable variability in supervision practices; and (5) a vulnerable and demanding student role. These themes are now described in more detail.

### Theme 1: variations in utility of pre-placement orientation and welcome at placement site

Receiving proper pre-placement orientation was emphasised as important and desirable by the participants. However, across the focus group interviews the participants’ views varied with regard to the relevance and adequacy of the information provided during the pre-placement orientation week.

There was an overload of information. I understand that the information can be relevant, but it is difficult to absorb it all. Some of the lectures and subjects was high flying, too in-depth and way over our head. (P1, FGA)

Overall, participants felt they were overloaded with oral and written information, which was difficult to absorb. Some participants also said that the utility of the obligatory pre-placement orientation information varied. The pre-placement orientation week was described by some participants as having long days, lacking interaction, and dialogue with the students. Some participants said they experienced that the utility was higher when they were gathered in smaller groups rather than plenary gatherings during the orientation week, as this allowed for more dialogue whereby it was easier to ask questions.

The day we were gathered in small student groups with our assigned nurse educator during the orientation week was vital for me. I experienced that day to be of most value – as it was easier to ask questions and interact. (P2, FGB)

Receiving more information, guidance and support during their orientation week concerning their obligatory written assignments as well as information about their assigned RN mentors was emphasised as important and desirable by the participants.

Practical information concerning the written self-assessment assignments was lacking. They just told us – this is the form you can use to evaluate your own performance and development. However, we have never written self-assessments before so many of us students did not understand what and how we should evaluate ourselves. (P4, FGA).

Moreover, participants had varied experience concerning the level of preparedness and the welcoming of students at the various placement sites. Some participants said that their placement site had arranged a welcoming milieu on their first day of placement whereas other participants told how they did not receive the same expected welcome from their placement site. Receiving a warm welcome was emphasised as important by the participants and was seen to have a positive influence on their first impression.

We received a nice welcome on our first day of placement. It was a nice feeling that you were expected. A nurse-coordinator was responsible for the reception/fixed day and gave us an introduction to the nursing home and the various wards. The only thing I missed was meeting/be introduced to my assigned nurse mentor. Only one mentor came and said hello to us on the reception day (P4, FGC).

### Theme 2: a challenging learning environment

Experiences and descriptions of a challenging learning environment was one of the major topics of discussion across all of the focus group interviews. Overall, participants spoke of the nursing home environment as instructive, even though they had difficulties elaborating on their specific learning outcome beyond learning fundamentals of care, interacting and communicating with older residents and, in particular, residents living with dementia. However, all participants referred to a challenging nursing home learning environment. The students shared several examples to illustrate this by emphasising factors such as lack of RNs available as mentors, lack of supervisory continuity, language difficulties, a mismatch between the RNs’ role and their first-year learning objectives, and a disparity between theory and practice.

Several participants described how sickness absence among the RN mentors led to lack of supervisory continuity. One student explained that she had to change placement site (e.g. nursing home ward) after 2 weeks on placement because her assigned RN mentor was off sick. Several participants voiced the need for ward managers to be better prepared by having a backup plan in case of sick leave among the RN mentors to ensure the predictability required by the participants.

We were four students on the ward, each assigned to an RN mentor. However, on the first day of placement we were told that three out of the four mentors were on sick leave – some with full or graded sickness absence. So, it was of course very challenging for some of us in the beginning of the placement period. (P4, FGA)

Another participant from another focus group said:

The placement site has agreed to have students. However, the RNs do not have time to supervise or follow-up on us as they are so busy with other responsibilities. It is a problem that there are so few available RNs in nursing homes. (P2, FGC)

Furthermore, some participants expressed that lack of supervisory continuity influenced their feelings of belongingness, security, and overall negative perspective of their placement experience. Additionally, participants raised concern about and questioned the RN mentors’ grounds for assessing the students’ performance and learning process because of deficiencies in continuity of supervision. Some participants described how they had very few working days together with their mentors prior to the mid-term assessment and between the mid-term and final assessment:

My RN mentor did not have grounds to say much during the mid-term assessment as we had not worked a lot of days together – for several reasons. It is busy you know, and my mentor needs to do her work with residents as well as dealing with me. So, I just needed to hang along. Besides, when I cared for residents in the morning, she [the RN mentor] was never present. So, how was she able to assess me and my progress in learning? (P4, FGB)

Moreover, participants across all focus groups reported a mismatch between the RNs’ role in nursing homes and the first-year students’ learning objectives. Some students experienced that the RNs had a more administrative role, focusing primarily on patient medication and performing more technical procedures. Several participants reported that their assigned RNs were less involved in the fundamentals of care in comparison with other healthcare workers.

We do not focus that much on medication nor administration during the first placement period. Our learning goals are more related to the fundamentals of care, learning to communicate and care for residents. If there only is one RN on the shift they are basically just running around with medications and performing RN required procedures. (P1, FGC)

However, a few students emphasised that even though they experienced that their RN mentor performed more administrative tasks and more technical procedures, they learned from that situation and valued experiencing the complexity surrounding patient care. Several participants across all focus groups expressed a disparity between theory and practice during their placement period. Many participants shared examples from daily nursing home practice whereby gaps from what they had learned in nursing school pre-placement were identified.

We realised quite quickly that everything is not done according to what we have learned in theory. (P1, FGC)

These gaps observed and reported by the participants were often related to aspects concerning hygiene and nursing documentation. Some participants considered nursing school to be too rigid and strict whereas other participants raised concern about how this disparity negatively influenced and shaped their practice. One student explained how she experienced that nursing documentation was not given priority on a busy workday. This influenced her own documentation practices:

The RN nurses are busy – there is not much time left to document nor update the residents care plans. And that has affected my practice as well – that I forget to document regarding the residents I have been involved with. When I perform interventions with residents, I know that I should document in the resident’s records. However, I have often experienced that when I get home, I remember that I have forgotten to document the care I have done, and I think the reason for that is that it is not part of the daily practice or not given sufficient prioritisation. (P2, FGA)

In response to this, participants in that focus group voiced the need for training related to the placement site’s electronic documentation systems.

Another issue that came up in two of the three focus group interviews was related to language difficulties associated with a multi-cultural workforce. A few participants experienced having RN mentors with linguistically diverse backgrounds. Some of those students shared examples of how language difficulties led to misunderstandings and frustration. One student voiced concern and questioned how linguistic challenges could potentially influence the quality of supervision, the outcome of the assessment, and their overall learning and placement experience.

When talking to my RN I experienced communication difficulties. When I asked specific questions, I sometimes experienced that my mentor gave answers on totally different things and subjects. I do not think my RN mentor always understood me, it was a bit frustrating both for me and for the residents as well. (P2, FGB)

Overall, participants emphasised gaining insight into the complexity and busyness of working as an RN in a nursing home during their placement period, recognising the RNs’ overall responsibility for residents while simultaneously mentoring students.

During the placement period we gradually gained a greater understanding of the busyness of the RN mentors, in fact how busy it really is working as a registered nurse in a nursing home. (P3, FGB)

### Theme 3: spending considerable placement time with non-registered nurses

Because of sick leave among RN mentors and the experienced mismatch between the RNs’ role and the first-year students’ learning objectives, several participants experienced being paired with a non-registered nurse over a long period of time during their placement period. Some participants expressed dissatisfaction with this arrangement because they missed being exposed to what some students referred to as nursing; On further exploration, this perception linked with their observed role of RNs in nursing homes related to performing more technical procedures.

I did get to see a lot of the fundamental of care when I worked alongside healthcare providers and non-registered nurses. And that is fine. However, it went almost 2 weeks between my shifts with my assigned RN mentor – so it took time before I learned what nursing was all about. (P4, FGA)

In contrast to more negative views, however, some participants expressed having more positive experiences working alongside non-registered nurses when their mentors were absent or busy. These students expressed spending more time with residents, interacting, and providing hands-on resident care when they worked alongside non-registered nurses. These students suggested that non-registered nurses were often more engaged in the fundamentals of care and (thus) this aspect of care provision. Hence, one participant emphasised that non-registered nurses could assume an important role in supervision during their placement period, as fundamentals of care was the main learning focus for first-year nursing students.

I have learned a lot by working alongside non-registered nurses – to see how they work. They [the non-registered nurses] have a lot of knowledge and skills. I have experienced that many non-registered nurses gave me many valuable advices. (P1, FGA)

However, this view was not shared by all participants because a majority emphasised that they received more valuable supervision and feedback when they worked with RNs in comparison with non-registered nurses. Furthermore, some participants expressed that non-registered nurses more often tended to view them as (purely) a supply of labour, which led some of the students to experience being exploited as manpower during their placement period.

Not all, but some of the non-registered nurses exploit us as pure supply of labour. They just tell us “go and do that and that” – handing over their work tasks on us students. Perhaps they are not familiar with what our student role implies when we are on placement? (P4, FGB)

### Theme 4: considerable variabilities in supervision practices

Participants across the focus groups described considerable variabilities in supervision practices from their assigned RN mentors and nurse educators overseeing their placement. Variability in supervision, support, follow-up, and feedback provided were reported. Some participants expressed having RN mentors who appeared motivated, engaged, and prepared for their supervisory role whereas other participants did not share the same opinion.

I have been lucky. I have had an RN mentor that’s really supported me during placements, cared for me, and teach me a lot. She [RN mentor] has been willing and interested in me and my learning process and arranged for me to learn and exposed me to various learning situations. (P4, FGB)

I also experienced that my RN mentor was really engaged in mentoring me during placement. In fact, my RN mentor came in on her day off and even when she was on sick leave to follow me up and take part in the meetings with the nurse educator. So, my mentor really put a lot of effort into me, ensuring that I had a good placement period. (P1, FGA)

In comparison, two students reported having fewer positive experiences.

Our RN mentor told us directly in the very beginning of our placement period that supervising students was the worst weeks of the year. So, you can easily say that it was not a flying start. (P1, FGB)

The participants who were satisfied with their supervision described RN mentors who guided them, spent time with them, explained, asked questions, listened to them, and were genuinely interested in aiding their learning process. Those students described how high levels of support enabled them to learn how to reflect on their own practice and work more independently throughout the placement period. On the other hand, those participants who had fewer positive experiences described mentors who appeared uncertain and uncommitted to their mentorship role, had high expectations of their skills, and were unfamiliar with the students’ learning objectives.

The mid-term assessment came as a surprise to my RN mentor. My mentor was not aware of my learning objectives, nor prepared and did not say much if anything during the assessment meeting. (P4, FGC)

I was a bit surprised that our mentor appeared so uncertain about what we students should do or what our learning objectives were – because I assume, they [the mentors] have some kind of course or receive information from the education institution. (P1, FGB)

Participants reported that the RNs’ supervisory style and degree and quality of feedback they received varied. Overall, participants called for more concrete feedback on their performance throughout their placement period. Some students experienced that it could be uncomfortable, leading them to be uncertain when being asked questions to justify their practice or knowledge. Some participants emphasised that this was particularly the case if feedback or questions were given or asked in the presence of residents or their next of kin. Most of the students said it was desirable to receive feedback and questions in private with their mentor.

I do prefer that we talk about things in private and not in front of residents. Then we can more openly discuss and reflect. I also feel more comfortable asking questions when it is not in front of residents – as that can be restrictive in respect of the resident. (P4, FGA)

Another student described how she experienced being scolded in the presence of a resident:

One time I experienced that a care assistant scolded on me in front of the resident. I did not understand what I had done wrong. But it was certainly not okay to be scolded in the presence of residents or in that way at all. It was so unpleasant and made me feel so uncomfortable and small. (P1, FGB)

Participants also described variability in the nurse educators’ approach regarding structure and content of supervision provided during their placement period. Some participants told how their nurse educator was present and available, and provided several supervisory meetings during the placement period allowing the students to meet and reflect on their placement experiences. Those students emphasised how such meetings with fellow students facilitated by the nurse educator were very educational. Other participants reported less structured supervision and more absence from the nurse educator responsible for overseeing their placement period. Furthermore, participants reported that nurse educators’ level of feedback (oral and written) content and demands on which emphasis was placed varied considerably in relation to the students’ written assignments as well as during the formal assessment meetings. The following statements across the three focus groups illustrate this variability.

We students talk amongst ourselves you know – and we realise that some nurse educators assign more tasks to some students than others and requires for example more written assignments beyond what’s expected of us or written in the course description. (P5, FGA)

The nurse educators place emphasis on different things and subjects, some are concerned with and question the students about theory and some teachers do not. They [the nurse educators] have different expectations and requirements. I heard a rumour about a teacher that failed several students on the mid-term assessment just because the students’ written self-assessment was not good enough – even though that has never been taught to us prior to placement [how to write self-assessments]. (P3, FGC)

The participants voiced concern and experienced such variabilities to be adverse, as they said it was unfortunate when nurse educators were not consistent in the requirements asked of the students.

### Theme 5: a vulnerable and demanding student role

Several participants across the focus group interviews reported feeling vulnerable during their placement period dealing with all-round pressure: having to deal with expectations from the placement site, being left with too much responsibility, academic demands (e.g. written assignments), and emotionally demanding placement experiences. Those participants having no prior exposure to patient care pre-placement reported this to a higher degree than those with prior patient experience.

Some participants reported feeling vulnerable when their RN mentor was absent or unavailable. In those situations, they said they were dependent on remaining staff to engage and take responsibility for students’ supervision. However, participants had variable experience with overall staff engagement and enthusiasm about having students on placement. Participants described both positive and less positive experiences related to inclusion in the workplace environment.

We students had to sit down during the morning report and just hope that someone took notice of us and said hello. Because we know that our assigned mentors have responsibility for us. However, when they are absent you rely on ward staff to engage. They are not committed or obliged in the same way – so you get the feeling that you sometimes are forcing yourself on others, feeling like a fifth wheel (on the wagon). (P3, FGC)

Participants gave server accounts of times when they were left to themselves in situations with residents which they were not comfortable, especially with complex cases and clinically ill residents. Being left with too much responsibility was reported as more challenging by some participants than by others, and more often by those students with no pre-placement clinical experience.

One time when we assisted a resident in need of nursing for the first time, we experienced that our RN mentor just left us to ourselves without saying anything – that was a bit scary as we did not know what do to with the resident. (P2, FGB)

I experienced that I suddenly was left all alone in the living room with several residents. Nobody had asked me if that was okay and if I was comfortable being there alone. Because I was not. There was a resident who was assigned to be guarded all the time due to turbulent behaviour and risk of falling. I was so nervous. I was left there without knowing what to do or how to reach other staff members. (P1, FGC)

Several participants expressed feeling great comfort and confidence in being paired with another student – and especially if the student with whom they were paired had pre-placement clinical experience. As one student said:

I experienced a lot of comfort being paired with and working alongside another student during placement. Everything felt easier and safer when we were two. The student I was paired with had prior experience with patient care – so it was easier to enter the residents’ room and interact with them when she (my fellow student) was there with me. We solved problems, discussed, reflected, and shared frustrations together. (P1, FGB)

Participants also voiced concern about the unequal power balance in the tripartite cooperation. A few students described how insufficient emphasis was placed on their voice during the assessment meetings when they, their assigned mentor, and nurse educator met to discuss their performance and learning process:

I am not sure if I should call it disagreement – but we (my RN mentor and I) had different perceptions and opinions about a situation. In that particular case, I experienced that the nurse educator sided with my mentor and was not really interested in what I had to say. So, I just had to agree with what was said during the assessment discussions – as there did not appear to be any room for discussion, misunderstandings, or frustration from my part. (P3, FGA)

Participants reported stress and high workloads during their placement period, especially concerning the expectation to take responsibility for their own learning. To document their own learning was something several students reported as difficult and challenging, representing a stressor during their placement period, especially as the student’s ability to self-reflect in writing was given considerable emphasis by the nurse educator in the formal assessment discussions. At the same time many participants said it was educationally instructive to write self-reflections. However, the participants voiced a need for more guidance and preparation prior to placement. Most of the participants stated that they were anxious or worried prior to placement because they were afraid of what everyone expected of them and their knowledge and technical skills.

I was really worried prior to the clinical placement period. I was so afraid that everyone expected that I had a lot of knowledge and could perform a lot of procedures. (P3, FGA).

## Discussion

The findings show that student nurses’ placement experience in nursing homes, not surprisingly and consistent with previous research [3, 10], varies with regard to which positive and less positive experiences were reported by the participants. Even though the participants in our study spoke of the nursing home placement as instructive, valuing their learning about the complexity of patient care, our findings call attention to and emphasise the characteristics of the nursing home context that need to be addressed to optimise the learning potential offered in these care settings.

Our findings indicate that staffing shortages and a general lack of available RN mentors, alongside mentors being absent through sick leave combined with their managerial role, introduced considerable vulnerability in student supervision, which negatively influenced students’ placement experiences. This vulnerability was manifested by supervisory discontinuity, changes of mentors, insufficient grounds for assessment, and students spending considerable placement time with non-registered nurses during their placement period. This is consistent with findings reported in a systematic review by Keeping-Burke and colleagues [3], who furthermore emphasise that such conditions lead to lack of role models, lack of feedback from RNs and, thus, missed learning opportunities [3]. Having the same RN mentor throughout the placement period has been reported by students to have a more positive influence on the supervisory relationship and the pedagogical atmosphere [26]. In comparison, students have reported frequent changes of mentors as stressful which, moreover, may impair their learning [27]. Thus, to enhance nursing homes as clinical placements and increase the capacity to foster positive placement and learning experiences, several have argued for development and application of placement models tailored to better accommodate the marginal nursing home context [28, 29]. Moreover, it is stressed that educational institutions should strive to be active agents of change and learning in the nursing home context precisely because the competence mix of staff is different from that of hospital settings [28].

Our findings also imply that the RNs’ role in nursing homes does not correspond sufficiently to the first-year students’ learning objectives that focus on the fundamentals of nursing care and associated skills. Indeed, it has been stressed that the RNs’ managerial role of working in nursing homes (e.g. the systematic shifts in the RNs’ role away from hands-on nursing to a more administrative focus) restricts students learning about gerontological nursing [17]. Hence, this mismatch may, moreover, influence and shape students’ perception of nursing or at the utmost lead to misconceptions about nursing pertaining more to technical skills. RN mentors represent powerful role models for student nurses in nursing homes where students mirror the attitudes and behaviours of their mentors [30]. One participant in our study reported her perception of nursing being more related to technical tasks rather than essential nursing skills based on that student’s observation. It is reported in other studies that student nurses view the nursing home as an environment that provides fewer learning opportunities compared with hospital settings, as students are concerned about “technical” care [3]. Hence, based on our findings and in line with Haugland and Giske [31], it is essential that first-year students see the enriched learning opportunities beyond task completion and develop a professional identity that promotes values embodied in the philosophy of person-centred care. Further research should therefore critically explore and extend our understanding of the implication of the RN’s role in a nursing home and the first-year student’s perception of nursing and the learning outcome.

Consistent with previous research [29], our findings suggest that the perception of feeling welcome varied. Feeling welcomed by the placement site and inclusion in the ward environment has been reported to be an important factor for students’ perceived success of learning and the overall quality of their placement experiences [32]. A positive and welcoming ward atmosphere and friendly, prepared, and available staff that provides high-quality care positively affected the quality of the learning environment [4, 6]. Moreover, some participants in our study reported that the degree of inclusion in the overall work environment during placement differed in ways which imply that the invitational qualities of the workplace were central to opportunities for learning afforded to students [33]. Certainly, clinical placement has great potential to enhance the experiences of the learners (e.g. students) as well as the wider organisation [34]. However, the readiness of nursing home staff and aptitude to engage and view students’ placement period as a learning activity likely depends on how well they and the workplace encourage staff to engage with these opportunities [35]. Hence, our findings imply that there remains room for improvement whereby nurse managers play an important role in efforts to enhance the nursing students’ learning environment in nursing homes [36]. However, there seems to be a lack of research on nursing home managers’ efforts in enhancing student nurses’ learning environment, a situation that warrants attention.

Our findings also indicate considerable variation in supervision practices provided by RN mentors, consistent with other studies exploring students’ experiences with nursing home placements [29]. Participants in our study reported that RNs’ motivation and enthusiasm for the mentorship role varied, alongside the level of feedback and support provided and knowledge and awareness of the students’ learning objectives, which all are factors that affect student learning [37, 38]. Likewise, the participants reported that the nurse educators’ supervisory approach, expectations, demands and, moreover, what they emphasised during the assessment discussions could vary, which was described by students as adverse. Indeed, previous research indicates that the nurse educator’s role in clinical practice is not clearly defined and varies by institution and country [39, 40]. In the United Kingdom (UK), national standards for the clinical education and mentorship have existed for many years [41, 42], and most recently these have been updated to reflect the shift in UK nursing from mentorship to supervision. Nevertheless, in Norway, as in most European Union countries, there are no specific educational requirements or national standards that guide the quality of clinical practice placements and mentoring practices [43]. Support from nurse educators with gerontological competence, solid orientation to the nursing home environment, and enthusiasm for aged care have been highlighted as central to enhancement of students’ learning and placement experiences, attitudes, and perceptions of aged care [5, 44, 45]. However, there exists a lack of nurse educators with expertise in gerontology [45], which is worrying and warrants attention. It has also been reported that students evaluate the role of the nurse educator as more important in their first year than in their final year of study [40], which implies that research into the nurse educator’s role regarding first-year students on nursing home placement is also warranted. Furthermore, creating national guidelines as part of a broader policy programme around quality clinical placement for students in nursing homes and other health services are required to enhance students’ educational experiences and to reduce the adverse variabilities experienced by student nurses.

Another noteworthy finding from this study is that language difficulties may be more profound during students’ placements within the nursing home context because of a more multi-cultural workforce. Some participants in our study reported communication difficulties with their RN mentors owing to language barriers, which are also reported in the study by Brynildsen et al. [29]. Successful communication between the students and their mentors plays an important role in satisfaction and the achievement of learning outcomes [46]. In Norwegian nursing homes immigrant nurses, often unfamiliar with the native country’s culture and history, can constitute up to 43% of the staff [8]. The importance of workforce diversity within healthcare systems to reduce healthcare disparities has been emphasised [47]. However, the implications (e.g. facilitators and barriers) of workforce diversity for effective mentorship practices of student nurses, and their learning outcome in clinical education, are not well documented and require further research, especially within the nursing home setting where the current knowledge base seems to be scarce or inadequate.

Finally, our findings call attention to the vulnerability associated with being a first-year student on placement in an unfamiliar and sometimes overwhelming nursing home context. Our findings indicate that students at times were left on their own with responsibility, with which they did not feel comfortable. Moreover, some participants reported being utilised as workers (used as an “extra pair of hands”) rather than learners by some staff members during their placement, consistent with existing literature [48]. The literature stresses that students must be provided with clear directions and expectations from nurse educators and nursing home staff to ensure their role as a learner rather than an independent worker during their placement period [3, 49]. Taking into consideration that nursing homes often represent students’ first placement and encounters with patient care [3], it is imperative to make this experience as enriched and positive as possible. This requirement is well documented as clinical placements, particularly in the initial periods, represent a stressful and emotionally challenging experience for these students [15], which if not mediated may impede learning and influence career choices and the decision to continue or drop out of the nurse education programme [50].

## Conclusions

The nursing home sector is predicted to grow in importance as a placement site for undergraduate students to meet the healthcare demands of an ageing population. This research provides insight into the contexts encountered by first-year students that seem to influence the quality of their placement experiences which may, moreover, prevent full utilisation of the learning potential offered in nursing homes. Findings reveal that the quality of pre-placement orientation and welcoming on placement varied. Moreover, students spend considerable placement time with non-registered nurses because of marginal access to RN mentors alongside the expressed mismatch between the RN’s role and the first-year student’s learning objectives. Consequently, this impedes access to important role models available to support the student’s growth and professional development. Furthermore, adverse variations in supervision and assessment practices were indicated and the vulnerability associated with being a first-year student emphasised. Hence, we propose that targeted efforts are warranted to foster positive placement experiences and enhance students’ clinical education in nursing homes.

### Limitations

This study has some limitations that merit consideration when interpreting the findings. First, the study was limited by a relatively small sample size conducted across three nursing homes within a Norwegian context that restricts the transferability of the findings. Nevertheless, the findings and issues raised are relevant in a national and international context especially for nursing education programs that apply the preceptorship model for clinical placement or similar teaching methods. Sample size and data saturation in qualitative research has been subject to enduring discussions arising from a variety of conceptual understandings [51]. The sample was highly specific for the aim of this study and the interview dialogue was strong, which enhances information power [52]. Furthermore, a high degree of consensus emerged during data analysis whereby themes were replicated across the dataset and deemed sufficient to satisfy the exploratory nature of this in-depth study [53].

Potential research biases should be acknowledged, given that the data collection and analysis were conducted by researchers with a nurse education background, which entails a prior understanding of the context. The researchers who conducted the focus group interviews were lecturers at the same educational institution as the students, which thus entails insider research from our own institutions [54]. However, the researchers were not involved in the students’ clinical placement period or had any prior professional relationship with the participants. Being interviewed by a lecturer might represent a bias because it may have influenced the participants to speak less freely than they might have done with an external interviewer. We tried to control for research biases by applying triangulation during the analysis process whereby four of the authors, two of whom were not involved in the interviews, actively participated in, and reflected upon the findings, providing a basis for checking interpretations to strengthen trustworthiness [55].

## Supplementary Information

**Additional file 1**. Focus group interview guide: Students.

## Data Availability

The datasets used and analysed during the current study are available from the corresponding author on reasonable request.

## Abbreviation

RN: Registered nurse
