# Supplementary material for: Nursing students’ experience with clinical placement in nursing homes: a focus group study
Source: BMC Nurs. 2021 Sep 6;20:159. doi: 10.1186/s12912-021-00690-4 (PMC8419895; doi:10.1186/s12912-021-00690-4)

**Focus group interview guide: Students**

**Opening question:**

How will you describe your overall experiences with clinical placement in nursing homes?

**Pre-placement orientation og preparation**

How will you describe the pre-placement orientation?

Alternatively: Suggestions for improvements?

**Reception on the placement site**

What characterized the first day on placement, the welcoming of students?

**Quality of supervision**

How will you describe the supervision and support you received from your assigned registered nurse mentor? /what characterized the supervision, support, and follow-up you received from your assigned registered nurse mentor?

What was your expectations concerning supervision from your assigned registered nurse and how were these expectations fulfilled?

How will you describe the supervision and support you received from your nurse educator? / what characterized the supervision, support, and follow-up you received from your nurse educator

What was your expectations concerning supervision from your nurse educator and how were these expectations fulfilled?

Alternatively: Suggestions for improvements?

**Tripartite Cooperation**

What characterized/how will you describe the tripartite cooperation during placement? (e.g. formal assessment discussions)

**Learning environment**

How will you describe the overall learning environment and your integration in the work environment?

How will you describe being a first-year student on placement in nursing home for the first time?

Alternatively: suggestions for improvement?

**Learning outcome**

What have your learned during your nursing home placement period?

What is important for you to be open to learning?

**Are there anything you would like to add, emphasis or comment on before we close? – any concluding remarks**


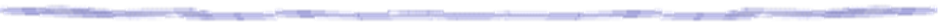

Supplement: Supplementary file 1 — Additional file 1. Focus group interview guide: Students. [file 12912_2021_690_MOESM1_ESM.docx]
